# Visual-auditory perception of prosodic focus in Japanese by native and non-native speakers

**DOI:** 10.3389/fnhum.2023.1237395

**Published:** 2023-09-21

**Authors:** Yixin Zhang, Xi Chen, Si Chen, Yuzhe Meng, Albert Lee

**Affiliations:** ^1^Department of Chinese and Bilingual Studies, The Hong Kong Polytechnic University, Hong Kong, Hong Kong SAR, China; ^2^Research Centre for Language, Cognition, and Neuroscience, Department of Chinese and Bilingual Studies, The Hong Kong Polytechnic University, Hong Kong, Hong Kong SAR, China; ^3^Research Institute for Smart Ageing, The Hong Kong Polytechnic University, Hong Kong, Hong Kong SAR, China; ^4^The HK PolyU-PekingU Research Centre on Chinese Linguistics, The Hong Kong Polytechnic University, Hong Kong, Hong Kong SAR, China; ^5^Department of Linguistics and Modern Language Studies, The Education University of Hong Kong, Hong Kong, Hong Kong SAR, China

**Keywords:** multi-sensory perception, visual-auditory integration, prosodic focus, focus perception, Japanese, Cantonese

## Abstract

**Introduction:**

Speech communication is multi-sensory in nature. Seeing a speaker’s head and face movements may significantly influence the listeners’ speech processing, especially when the auditory information is not clear enough. However, research on the visual-auditory integration speech processing has left prosodic perception less well investigated than segmental perception. Furthermore, while native Japanese speakers tend to use less visual cues in segmental perception than in other western languages, to what extent the visual cues are used in Japanese focus perception by the native and non-native listeners remains unknown. To fill in these gaps, we test focus perception in Japanese among native Japanese speakers and Cantonese speakers who learn Japanese, using auditory-only and auditory-visual sentences as stimuli.

**Methodology:**

Thirty native Tokyo Japanese speakers and thirty Cantonese-speaking Japanese learners who had passed the Japanese-Language Proficiency Test with level N2 or N3 were asked to judge the naturalness of 28 question-answer pairs made up of broad focus eliciting questions and three-word answers carrying broad focus, or contrastive or non-contrastive narrow focus on the middle object words. Question-answer pairs were presented in two sensory modalities, auditory-only and visual-auditory modalities in two separate experimental sessions.

**Results:**

Both the Japanese and Cantonese groups showed weak integration of visual cues in the judgement of naturalness. Visual-auditory modality only significantly influenced Japanese participants’ perception when the questions and answers were mismatched, but when the answers carried non-contrastive narrow focus, the visual cues impeded rather than facilitated their judgement. Also, the influences of specific visual cues like the displacement of eyebrows or head movements of both Japanese and Cantonese participants’ responses were only significant when the questions and answers were mismatched. While Japanese participants consistently relied on the left eyebrow for focus perception, the Cantonese participants referred to head movements more often.

**Discussion:**

The lack of visual-auditory integration in Japanese speaking population found in segmental perception also exist in prosodic perception of focus. Not much foreign language effects has been found among the Cantonese-speaking learners either, suggesting a limited use of facial expressions in focus marking by native and non-native Japanese speakers. Overall, the present findings indicate that the integration of visual cues in perception of focus may be specific to languages rather than universal, adding to our understanding of multisensory speech perception.

## 1. Introduction

Face-to-face communication among human beings is multi-sensory in nature. Spoken messages are not only encoded by linguistic and para-linguistic information but also transmitted through visual mode. It is well-established in the literature that seeing a speaker’s head and face movements may significantly influence listeners’ speech processing, especially when auditory information is damaged in quality or presented in noisy environments (Sumby and Pollack, 1954; McGurk and MacDonald, 1976; Kohlrausch and van de Par, 1999; Bertelson et al., 2000; Davis and Kim, 2006). The existing experiments on visual-auditory speech processing mainly investigate the enhancing or misleading effects of visual cues on the perception of phonemes (i.e., McGurk Effects, McGurk and MacDonald, 1976) or emotional and attitudinal information. The integration of multi-sensory information in prosodic perception, however, remains to be explored, especially from a cross-linguistic perspective.

Prosody is an important perceivable integral clue of speech that plays a critical role in signaling lexical stress and encoding information structure on the utterance level (Domínguez et al., 2014). Human beings organize their speech according to their communication purposes. Such organization is referred to as information structure (cf. Lambrecht, 1996). An important approach to investigate information structure is to distinguish between the topic and the focus, that is, between the background information and the information highlighted (Lambrecht, 1996; Arnold et al., 2013). In a complete discourse, the information on focus can either be the entire sentence or a specific part of it, which are usually referred to as broad focus and narrow focus (Halliday, 1967). The narrow focus used to correct or reject the inappropriate information of speech is also known as contrastive focus. In many languages, focal prominence is marked by observable acoustic features that make certain constituents stand out from other surrounding elements in utterances, and henceforth we will use “(linguistic) prominence” to refer to such phenomenon, whether it is at lexical or phrasal levels (cf. Cangemi and Baumann, 2020). The acoustic features, despite language-specific variations, include longer duration, larger intensity, special pitch patterns and voice quality (cf. Gussenhoven and Chen(eds), 2021).

Visual cues like eyebrow movements, exaggeration of articulatory movements and head movements have been found to correlate with the acoustic features that mark prominence (Graf et al., 2002; Dohen et al., 2004; Cho and McQueen, 2005; Beskow et al., 2006; Scarborough et al., 2009). In perceptual studies, by varying pitch accents and eyebrow movements using synthetic variation, Krahmer et al. (2002) found that both cues have a significant effect on prominence perception, though pitch accents had a much larger effect than eyebrows. By using real person videos, Swerts and Krahmer (2008) found that auditory-visual congruence enhanced prominence perception while incongruence hindered it; they argued for relatively equal weightings of the auditory and the visual stimuli.

In addition, different facial parts are of different weightings in prominence marking and perception, though the exact effects are yet to be determined. A number of studies using synthetic animation to systematically vary visual stimuli show that head and eyebrow movements (e.g., a head nod and/or an eyebrow raise) alone can serve as cues to prominence in perception, even when auditory cues are absent. As early as, Granström et al. (1999) found a general coupling between eyebrow raising and perceived prominence and a tight perceptual connection between eyebrow movement and certain f0 contours in Swedish. House et al. (2001) found that both eyebrow and head movements are powerful visual cues for prominence perception on their own by varying head and facial movements over the same Swedish speech stimuli. By contrast, Scarborough et al. (2009) analyzed the videos from three English speakers and found larger and faster facial movements when constituents carrying a lexical stress or a phrasal focus were produced; the follow-up perceptual experiment of the silent videos showed that phrasal focus was better perceived than lexical stress and the primary cue was chin opening rather than head or eyebrow movements. Swerts and Krahmer (2008) argued that the disparities between Scarborough et al. (2009) and other studies might stem from differing methodologies. Specifically, Scarborough et al. (2009) employed real person videos, whereas other studies used synthetic animation, where variation may be amplified due to the impact of early observational studies. However, by using real person videos with manipulation of showing the entire or partial faces themselves, Swerts and Krahmer (2008) still found that it was the upper and left facial area that weighed greater than the lower and right facial area in prominence perception. The weighting of facial parts in prosody perception is of particular interest in the recent years as people are covering their lower facial parts with masks due to the COVID-19 pandemic. To what extent the upper facial areas alone could be a cue for prosodic prominence, and whether there would still be an asymmetry between the left and right eyebrows in signaling prosodic prominence are worth further research.

Whether the integration of auditory and visual cues in prominence perception varies across languages has not been investigated because the existing studies have predominantly focused on European languages. However, it has been established that McGurk Effects are language-specific, and emotional and attitudinal perception are largely influenced by culture (Ekman et al., 1987; Matsumoto and Ekman, 1989; Matsumoto, 1992; Biehl et al., 1997). A non-western language, Japanese, for instance, shows much weaker McGurk Effects than western languages. Through a series of experiments, it has been demonstrated that Japanese speakers rely little on visual cues in normal situations; instead, they only incorporate the visual information when the background is noisy or speakers deliberately prolong vowels or exaggerate lip movements (Sekiyama and Tohkura, 1991; Sekiyama, 1994; Sekiyama et al., 1995). The authors attributed the weaker McGurk effects found in Japanese to the differences in phonological complexity between Japanese and English and correspondingly, the lack of visually distinct phonemes and consonant clusters in Japanese (Sekiyama and Tohkura, 1993). Cultural aspects of Japan, such as cultural conventions against direct gaze and subtler facial expressions, may also decrease native Japanese speakers’ reliance on visual cues for speech perception (Matsumoto and Ekman, 1989; Matsumoto, 1992; Sekiyama and Tohkura, 1993; Sekiyama, 1994).

In addition, the linguistic background of listeners also affects their integration of visual and auditory cues in speech perception, though the existing studies have focused on segments and lexical prosody rather than focal prominence on the utterance level. McGurk Effects are found to be enhanced in foreign language perception, namely, listeners tend to use more visual cues in processing non-native language compared to their native language. Such enhancing effects are found cross-linguistically in Japanese, American, Chinese, Spanish and German listeners who perceive foreign languages (Fuster-Duran, 1996; Chen and Hazan, 2007; Sekiyama and Burnham, 2008). Studies on suprasegmental perception focusing on pitch contours, by contrast, found an integration of visual and auditory cues in tone perception. For instance, the series of studies conducted by Burnham et al. (2001a,b, 2022) showed that not only native Cantonese speakers use visual cues in lexical tone perception, but non-Cantonese speaking participants that speak tone (e.g., Thai) or non-tone languages (e.g., English) are able to draw on visual information in an AB discrimination task of Cantonese tones as well. In terms of the existence of visual information for tone, the authors summarized that it “is available in the absence of experience with the language in question, and even in the absence of experience with the lexical use of tone” (Burnham et al., 2001b). Nevertheless, Burnham et al. (2022) did not analyze in detail which visual cues were used by the listeners in tone perception. Burnham et al. (2022) only demonstrated that tone discrimination was facilitated the most by head and face cues, followed by head-only and face-only cues. The connection between facial movements and pitch observed in speakers with different language backgrounds suggests that such connection may also commonly exist in focal perception. Then, it becomes an interesting question of whether the integration of visual and auditory cues might be amplified by foreign language effects, particularly when listeners have experience with tonal languages.

Prior research established the necessity to compare two subsets of Asian speakers: Cantonese and Japanese speakers. The McGurk effect refers to the phenomenon in which conflicting auditory and visual cues produce a unique perception. This effect was originally observed among English-speaking participants (McGurk and MacDonald, 1976), and has then primarily been explored in English-language studies. While this phenomenon exists beyond English, its manifestation varies across languages (for Japanese, see Sekiyama and Tohkura, 1991; Sekiyama and Tohkura, 1993; for Mandarin, see Sekiyama, 1997; for Kurdish, see Asiaee et al., 2016). For instance, studies have indicated that Japanese speakers utilize visual information less than English speakers, leading to a distinctive “Japanese McGurk” effect (Sekiyama and Tohkura, 1991, Burnham et al., 2022). This reduced visual dependency can be linked to cultural conventions against direct gaze (Sekiyama and Tohkura, 1993; Sekiyama, 1994) and the lack of visually distinct phonemes and consonant clusters in Japanese (Sekiyama and Tohkura, 1993). Further, cultural aspects of Japan, such as subtler facial expressions, could decrease native Japanese speakers’ reliance on visual cues for speech perception (Matsumoto and Ekman, 1989; Matsumoto, 1992). Conversely, studies showed that Cantonese speakers do incorporate visual cues in lexical tonal perception (Burnham et al., 2001a,^2022^), indicating how cultural and linguistic factors can influence visual cue use. In comparing these groups, we scrutinized how varied cultural norms and linguistic structures within the same broad geographical region affect prosodic focus perception. This comparison offers a more intricate understanding of the relationship between culture, language, and speech perception. To fill in the aforementioned research gaps, we tested focus perception in Japanese among native Japanese speakers and Cantonese-speaking learners of Japanese, using auditory-only and auditory-visual sentences as stimuli. For visual cues, we presented only the upper faces while the lower faces were covered with masks.

The distinctiveness of the Tokyo Japanese language lies in its use of a mora-based rhythm [mora is defined as “a unit of duration” by Bloch (1950)], impacting the assignment of lexical pitch accent. This pitch accent, referring to an abrupt fall in f0 from the accented vowel to the unaccent vowel within a word (Kawahara, 2015). A difference in meaning between two words can be illustrated through the presence or absence of a pitch accent. This can be exemplified by the words “雨が” (rain, /a’mega/) and “あめが” (candy, /amega/), where the former contains a pitch accent — indicated by the symbol /’/ — which signifies the presence of the accent, while the latter does not. An accented vowel is assigned by a H(igh) tone, followed by a L(ow) tone on the succeeding vowel. This sequence results in a sharp H-L fall in f0, a phenomenon not observed in unaccented words. Furthermore, Japanese differentiates words not only by accent presence but also by its position in a word. This accent assignment pattern is encapsulated by the “n+1” rule, as referenced in multiple studies (Kubozono, 2012; Ito and Mester, 2016; Haraguchi, 2017). In words comprising n syllables, there are n+1 accentual patterns. This n+1 accent patterns, as illustrated by Kawahara in 2015, can manifest as: a. initial accent (life, /i’noti /); b. second accent (heart, /koko’ro/); c. final accent (head, /atama’/); or d. unaccented (city, /miyako/). For these examples, the corresponding tone-spreading patterns are HLL for a, LHL for b, LLH for c, and LHH for d (here, d is typically employed for a triphthong noun).

Research on prosodic marking of focus in Japanese generates complex results. On the one hand, several acoustic studies showed that narrow focal prominence in Japanese is marked by relatively complex mechanisms, including local f0 range expansion, an increase in f0 and intensity, prominence-lending boundary f0 movements, and post-focus compression (i.e., post-focus constituents are reduced in f0 and intensity) but not changes in word accent (e.g., Venditti et al., 2008; Ishihara, 2016). Nevertheless, word- and utterance-level prosodies did interact with each other, as Lee and Xu (2012) only found post-focus compression of f0 range in accented stimuli. On the other hand, Mizuguchi and Tateishi (2020, 2023) argued that the prosodic cues in Japanese were not strong enough to mark utterance-level focus; in particular, f0 is not a prominent cue that signals focal prominence on utterance level. To be specific, Mizuguchi and Tateishi did find that Japanese speakers used prosodic cues to signal contrastive focus. However, they did not find much statistical significance between contrastive focus and broad focus in the acoustic analyses. In their follow-up perceptual studies, only when a word carrying contrastive focus preceded or followed an unaccented word were the differences between narrow and broad focus significant. Additionally, a study examining the realization and perception of focus in Tokyo Japanese phone numbers found that, even when accentual peaks were assigned, the marking of contrastive focus is relatively weak (Lee et al., 2018). These results raise intriguing questions regarding the integration of visual cues in focus perception in Japanese. It is plausible that the observed lesser usage of visual cues in perception of segmentals also applies to Japanese speakers’ prosodic perception. Conversely, the complicated and potentially weak prosodic marking of focus might prompt Japanese speakers to lean more on the ties between f0 and facial movements and to increase the processing of visual cues. These hypotheses need to be further explored.

**FIGURE 1:**
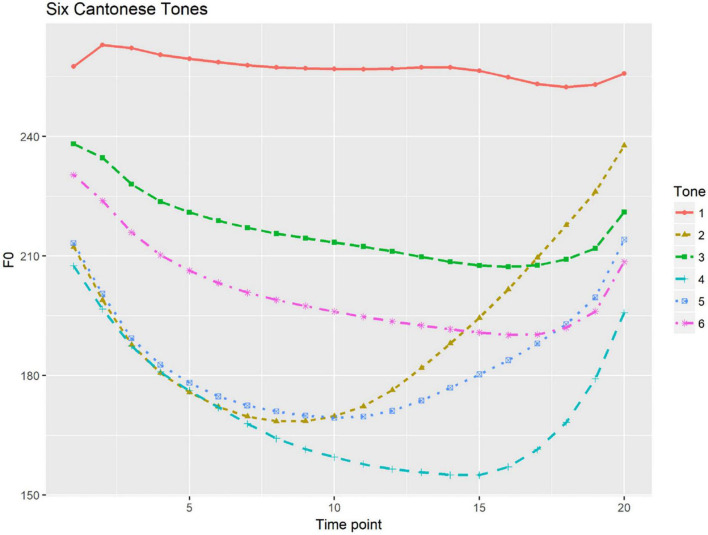
Six Cantonese tones with time-normalized mean f0 contours calculated speech production by seven female speakers [illustrated from Chen et al. (2022)].

Compared to the two pitch-accent patterns that Japanese features, Cantonese has a complex tone system of six full tones (full tones: /si1/ “silk,” /si2/ “history,” /si3/ “try,” /si4/ “time,” /si5/ “market,” /si6/ “thing”) (cf. Bauer and Benedict, 1997). Figure 1 presents the six tonal contours of Cantonese, each of which has been time-normalized and produced by seven female native speakers, as depicted by Chen et al. (2022). The six tones are High level tone (Tone 1); Mid rising tone (Tone 2); Mid level tone (Tone 3); Low falling tone (Tone 4); Low rising tone (Tone 5); and Low level tone (Tone 6). F0 is the primary acoustic cue in Cantonese tone perception both in real speech (Fok Chan, 1974) and synthesized speech (Vance, 1977). Cantonese does not have lexical stress but does mark focal prominence at the utterance level. Cantonese focus prominence is primarily marked by an increase in duration and intensity (Wu and Xu, 2010; Fung and Mok, 2014, 2018). With regard to f0, narrow focus can trigger on-focus f0 expansion but the expansion is influenced by the lexical tones of the on-focus components (Gu and Lee, 2007; Wu and Xu, 2010), while whether there is a notable post-focus compression in Cantonese remains controversial (cf. Wu and Xu, 2010; Wu and Chung, 2011). Perceptually, an increase in duration has been found to be the primary cue to focus, while the roles pitch movements play are specific to the lexical tones carried by the components on focus (Leemann et al., 2016; Fung and Mok, 2018).

Based on the previous studies, we raised three specific research questions and made the corresponding hypotheses: (1) Are visual cues integrated by the native and non-native speakers in the perception of focal prominence in Japanese? (2) What auditory and visual cues are critical in the perception of prosodic prominence by native and non-native Japanese speakers when both cues are presented? (3) Whether and how is focus perception influenced by changes in different facial parts among native and non-native Japanese speakers when both auditory and visual cues are presented?

For each research question, we also made corresponding hypotheses. Firstly, both native and non-native listeners will use visual cues in the perception of focal prominence in Japanese, but the Cantonese listeners will rely more on visual cues than native Japanese listeners (H1). This hypothesis is made due to the widely reported connection between pitch and facial movements. At the same time, being a foreign language learner may motivate the Cantonese listeners to use more visual cues (i.e., foreign language effects). In addition, since Krahmer et al. (2002) found auditory cues to be more important than eyebrow movement in the perception of pitch accent, we predicted that auditory cues will be more important than visual cues, though there may be some differences between the native and non-native listeners (H2). Finally, based on the findings of Krahmer and Swerts (2007), it is hypothesized that eyebrow raise, especially, the left eyebrow raise, is of the greatest weighting in signaling focal prominence (H3).

## 2. Methodology

### 2.1. Stimulus preparation

The auditory stimuli used in this study comprised 28 three-word Japanese sentences, half of which contained accented words and the other half, unaccented words (for examples see Table 1). For instance, the sentence “小山さんはサラダを残した” (Mr. Koyama left the salad) contains the accented word “サラダ (salad, /sa’lada/),” and the sentence “村田さんは畳を拭いた” (Mr. Murata wiped the tatami) includes the unaccented word “畳 (tatami, /tatami/).” For accented words, there is a high pitch that starts from the first mora and continues up to the accent, followed by a sharp drop on the next mora. Conversely, unaccented words exhibit a consistent high pitch across all morae. We intentionally chose simple words and sentence structures to ensure that the placement of accents was clear and unambiguous. The naturalness and grammaticality of all adopted materials were approved by four native Japanese speakers who did not participate in the perceptual experiment.

**TABLE 1:** Examples of stimuli.

Accented-word-sentence	小山さんはサラダを残した。 Mr. Koyama left the salad.
Unaccented-word-sentence	村田さんは畳を拭いた。 Mr. Murata wiped the tatami.

Two native female Tokyo Japanese speakers with college degrees (aged 33 and 35) were invited to make the recording for the perception experiment. The two female speakers were relatively new residents of Hong Kong, each having lived there for less than a year. The speakers were recorded simultaneously in a soundproof language laboratory at the Hong Kong Polytechnic University. A microphone with a sampling rate of 44.1k Hz and one SONY Alpha 7 camera was positioned 0.75 meters in front of the two speakers. Natural production of stimulus answers with broad focus as well as contrastive narrow and non-contrastive focus on the second word (i.e., the object) was elicited through semi-spontaneous dialogues over randomized pictures. Stimulus pictures were displayed on a computer screen (1920 × 1080) with E-prime 3.0 (Psychology Software Tools Inc, 2016, Pittsburgh, PA). To provide a more comprehensive understanding of our experimental procedure, we have included all images used to elicit stimulus responses in the Appendix. The speakers were asked to look straight at the camera when speaking, and both of them wore facial masks. The audio and video recordings were synchronized using *Adobe Premiere Pro* (Adobe Premiere Pro Help, n.d.), and the audio recordings were segmented in Praat (Boersma and Weenink, 2018) for subject, object, and verb in each sentence. In total, 84 stimulus answers [14 sentences × 2 word accents (accented and unaccented) × 3 focus conditions (broad, contrastive and non-contrastive)] and nine filler answers were recorded. The fillers were combined with five eliciting questions that elicited different types of focus.

For the perceptual experiment, auditory-only and auditory-visual conditions were created. Within each condition, matched and mismatched question-answer pairs were used. Since the paper mainly aims to test the effect of group, accentedness of words, integration of visual information in processing focus, given the lengthy experimental sessions which involved a total of 228 trials across both modalities, we chose to control the questions to be those elicit broad-focus and the answers to have either broad, narrow or contrastive focus. The matched pairs were therefore made up of broad-focus-eliciting questions and broad-focus answers (BQBA), and the mismatched pairs were made up of a broad-focus-eliciting question and a contrastive narrow focus (BQCA) or a broad-focus-eliciting question and a non-contrastive narrow focus (BQNA) (for examples see Table 2). These selections were strategically made to enhance the acoustic prosodic distinction between pairs. A question eliciting a narrow focus will have answers with broad and contrastive focus as the mismatched pair and answers with the narrow focus as the matched pair. In our acoustic analysis and in line with a previous research (Pictures used to elicit natural production from the two speakers were provided as Supplementary material, e.g., Hwang, 2011), contrastive and narrow focus were found to have relatively subtle differences when perceived auditorily. By choosing broad-focus questions to pair with either contrastive or narrow focus answers, we made sure that the differences in prosodic acoustic features were more salient between matched and mismatched pairs. Future studies may explore the effects of other types of focus-eliciting questions to test if the findings may hold across questions with various focus types. In total four subgroups of stimuli were created:

**TABLE 2:** Examples of Q&A pairs for the four subgroups.

(1) VA-BQCA (4) AO-BQCA	Matched pairs	Q: 何かあったの? What happened? A: [久保田さんは本を取った。]_FOC_ [Mr. Kubota took a book.] _FOC_
	Mismatched pairs	Q: 何かあったの? What happened? A: 久保田さんは[本]_FOC_ を取った。 Mr. Kubota took a [book]_contrastive focus_.
**(2)** VA-BQNA (3) AO-BQNA	Matched pairs	Q: 何かあったの What happened? A: [久保田さんは本を取った。]_FOC_ [Mr. Kubota took a book.] _FOC_
	Mismatched pairs	Q: 何かあったの What happened? A: 久保田さんは[本]_FOC_ を取った。 Mr. Kubota took a [book]_narrow focus_.

VA-BQCA: *Visual-auditory* stimuli with matched and mismatched pairs; mismatched pairs invoke answers carrying contrastive narrow focus.

VA-BQNA: *Visual-auditory* stimuli with matched and mismatched pairs; mismatched pairs invoke answers carrying non-contrastive narrow focus.

AO-BQCA: *Audio-only* stimuli with matched and mismatched pairs; mismatched pairs invoke answers carrying contrastive narrow focus.

AO-BQNA: *Audio-only* stimuli with matched and mismatched pairs; mismatched pairs invoke answers carrying non-contrastive narrow focus.

### 2.2. Participants

Thirty native Japanese speakers and 30 Cantonese-speaking Japanese learners were recruited. Japanese participants (JP) were all born and raised inside Greater Tokyo, speaking native Tokyo Japanese (Table 3). The majority of JP could not speak, read, listen, or write any tonal language, except that three participants claimed that their Mandarin was at an elementary level. Cantonese-speaking Japanese learners (CA) were native Hong Kong Cantonese speakers who had passed the Japanese Language Proficiency Test with level N2 or N3. The JLPT is a standardized criterion-referenced test to evaluate and certify Japanese language proficiency for non-native speakers (Japan Language Testing Center, 2012). The N2 level signifies that the learner can comprehend everyday topics, read and understand passages on familiar matters and follow both written and spoken discussions on specialized subjects; The N3 level certifies that the learner has the ability to understand Japanese used in everyday situations to a certain degree. Out of these 30 CA learners, only 2 had not previously visited Japan. The remaining 28 participants have, on average, spent approximately 24.18 days (standard deviation: 17.44 days) in Japan. Furthermore, these participants had been learning Japanese for an average of 3.98 years, with a standard deviation of 2.29 years. Participants were controlled musical backgrounds, and none of them had perfect pitch or reported any problems in reading, hearing, and speech.

**TABLE 3:** Information of participants.

	Age ± SD	Male/Female	Musically trained/not musically trained	Years of musical training	Played instrument/Sung in the past 5 years
JP	25.73 ± 3.96	14/16	21/30	7.09 ± 4.76	2/30
CA	22.70 ± 3.54	14/16	18/30	7.44 ± 5.15	3/30

### 2.3. Procedure

**FIGURE 2:**
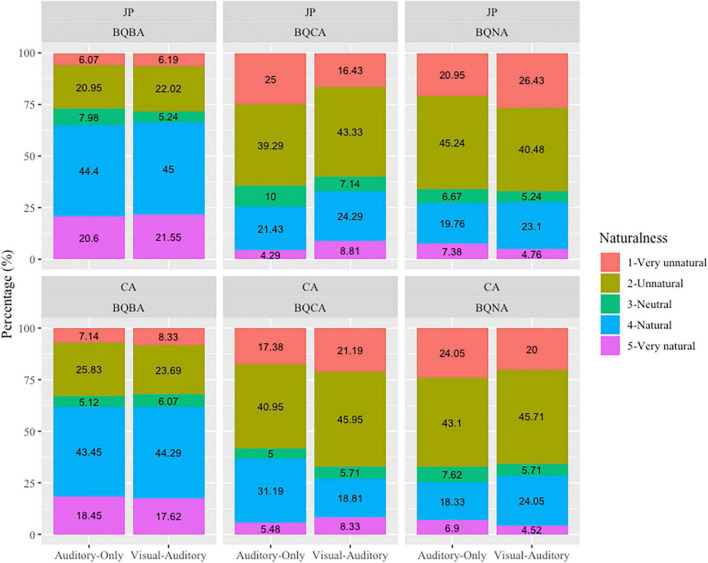
5-Likert scale for the response page.

The perception experiment was designed using PsychoPy (Peirce, 2009) and conducted online through the Pavlovia online platform because the speech lab was closed during the pandemic. Before the experiment, all participants were asked to pass the Huggins pitch-based earphone detection test (Milne et al., 2021) to determine whether they were wearing earphones or headphones during the online experiment. In each trial, the participants were first presented with the question-answer pair and then they were required to judge the naturalness of the dialogue by pressing 1 to 5 on their keyboards according to the scale in Figure 2. Participants were allowed to take a break anytime as needed.

Two practice sessions consisting of six matched and six mismatched trials were given to the participants before the formal experimental session, each made up of auditory-only or auditory-visual stimuli. Matched trials consisted of narrow-focused Q-A pairs of objects and verbs for subgroups (1) and (3) and contrastive-focused Q-A pairs of objects and verbs for subgroups (2) and (4). Mismatched trials were combined with narrow focus eliciting questions on objects or verbs and broad-focused answers for subgroups (1) and (3), with contrastive focus eliciting questions on objects or verbs and broad-focused answers for subgroups (2) and (4). The sentences used in the practice sessions were all selected from fillers. Participants need to correctly answer more than 7 trials in the practice session to enter into the formal experiment. We did not discard any participant’s data because they all passed the headphone detection test and practice session with >50% accuracy.

Two female research assistants who were trained in linguistics monitored the practice session via video calls. The participants were left on their own during the formal experiments. Each experimental session contains 114 trials (14 Sentences 2 Focus Types 2 Accent types + 58 Fillers), lasting for about 2 h in total. A simple three-choice question on the content of the dialogue (e.g, “Who took a book in the last recording?” for the example sentence in Table 3) popped up after every ten trials to help participants focus on the task. A warning would be shown on the screen to draw the participants’ attention if they made an incorrect choice.

Within each language group, half of the participants were tested with stimulus subgroup (1) and (4) and the other half with (2) and (3). Participant groups and subgroups were matched in gender and age. The order of participants perceiving subgroups (1) and (4) or (2) and (3) was balanced within each group, that is, half of the subjects who perceived the same subgroups [e.g., (1) and (4)] listened to the audio-only stimuli first while the other half started with the audio-visual stimuli first.

### 2.4. Data analysis

Answers for matched (i.e., broad focus) and mismatched (i.e., contrastive and non-contrastive narrow focus) trials were pre-processed differently because for the former, “5” was the best answer and “1” was the worst answer, whereas for the latter, “5” was the worst answer and “1” was the best answer. To facilitate statistical analysis, the answer for mismatched trials were subtracted by 6, making “5” the best answer for the whole data set. Correspondingly, in matched trials with answers involving broad focus, the higher the scores are, the more natural the question-answer pairs are; in mismatched trials with answers involving contrastive and non-contrastive narrow focus, the higher the scores are, the less natural the question-answer pairs are.

Cumulative Link Mixed Models (CLMMs) were established to evaluate the effects of different factors on the ordinal response made by participants as CLMMs make it possible to analyze ordinal response variables while allowing the use of random effects (Christensen and Brockhoff, 2013; Christensen, 2019). A CLMM for an ordinal variable, Yi that can fall in J categories is a linear model for a transformation of cumulative probabilities, γi j through a link function:


(1) P(Yi≥j)=γij=F(θj-xTiβ)i=1,…,n;j=1,…,J

where the intercept parameters


(2) -∞≡θ⁢0≥θ⁢1≥…≥θ⁢J-1≥θ⁢J≡∞

In this model, j is the level of an ordered category with J levels, and i is the number of independent variables. In the present study, *j* = 1 refers to “1 - the worst answer”… and *j* = 5 refers to “5 - the best answer” while i refers to the proposed fixed effects.

To test the first hypothesis, a CLMM was fitted to test the effects of Sensory Modality (AO vs. VA), Participant Group (JP vs. CA), Focus (Broad vs. Non-contrastive Narrow vs. Contrastive), Pitch Accent (Accented vs. Unaccented) and their interactions on participants’ judgmentof naturalness of both auditory-only and visual-auditory stimuli. Participants and stimuli were selected as the random effects according to previous studies (Jaeger, 2008; Barr et al., 2013). Both forward selection and backward elimination methods are often used in fitting regression models and both of them have their own advantages and disadvantages (Chowdhury and Turin, 2020). We used forward selection because this method starts with a smaller model compared to the backward elimination methods and this method may be less susceptible to collinearity. Tukey *post-hoc* test was used to assess the significance of differences between pairs of group means.

To test the last two hypotheses, CLMMs were further fitted for each participant’s group and each focus type, using acoustic and visual cues and visual cues only as fixed effects, respectively. The optimal fixed structure of each model was selected by stepwise comparisons by adding each variable from the simplest structure to the most complex. The likelihood ratio tests were used to compare different models to determine whether including factors from the analysis led to a better fit (Field et al., 2012). All the CLMMs were established using clmm in the Original package (Christensen, 2019) in R (R Core Team, 2022). The mathematical interpretation of CLMMs was presented in the appendix.

Specifically, acoustic cues included duration, mean f0, f0 range (i.e., the difference between the maximum and the minimum f0), mean intensity, intensity range (i.e., the difference between the maximum and the minimum intensity), the maximum and minimum f0 difference between two neighboring time points, the maximum and minimum intensity difference between two neighboring time points of the on-focus object syllables, the post-focus verb syllables and the pre-focus subject syllables. The constituents of sentences — the subject, object, and verb — were individually segmented using Praat (Boersma and Weenink, 2018). Following the segmentation, we utilized the Praat script ProsodyPro (Xu, 2013) to derive the values for f0, intensity, and duration from each sentential component. All ranges for f0 and intensity were calculated after applying an outlier exclusion procedure for f0 and intensity values based on interquartile ranges (IQR). Specifically, any data points falling outside the range defined by the 25th (Q1) and 75th (Q3) percentiles were considered outliers and were excluded. The formula we used for outlier removal was: [Q1 - 1.5IQR, Q3 + 1.5IQR], where IQR = Q3 - Q1. All these parameters were calculated, respectively for subjects, objects and verbs, which were also pre-, on- and post-focus words in mismatched trials with contrastive and non-contrastive narrow focus.

**FIGURE 3:**
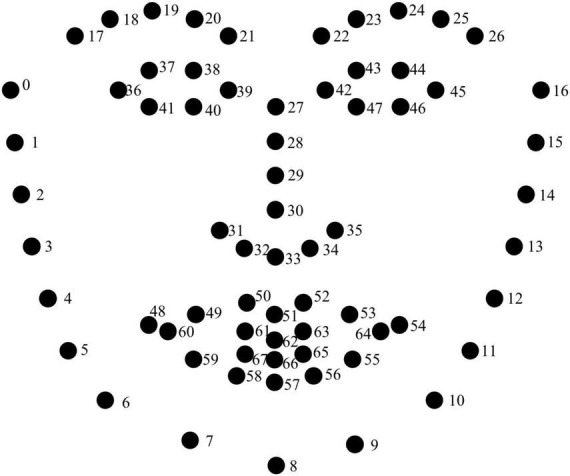
The distribution of facial points in OpenFace.

Visual cues include left and right eyebrows and head displacements and the velocities of movements, which were computed using real-time facial kinematics data extracted from OpenFace 2.0, a facial behavior analysis tool kit to track the points of interest (Figure 3). Our points of interest were Points 22-26 (i.e., the left eyebrow) and Points 17-21 (i.e., the right eyebrow) and Point 27 (i.e., the head). All displacement measurements were first normalized by dividing the maximum distance value from the nose root to the left and right cheek edges (the maximum value in Figure 3 between points 0 to 27 and points 16 to 27). After normalization, the following parameters of the on-focus periods were calculated: maximum displacements of the left eyebrows, the maximum displacements of the right eyebrows, the leftwards and rightwards maximum displacements of the head, the upwards and downwards maximum displacements of the head, the maximum velocity of the left eyebrows, the maximum velocity of the right eyebrows, the leftwards and rightwards movement maximum velocity of the head and the upwards and downwards movement maximum velocity of the head.

## 3. Results

### 3.1. Multisensory focus perception

**FIGURE 4:**
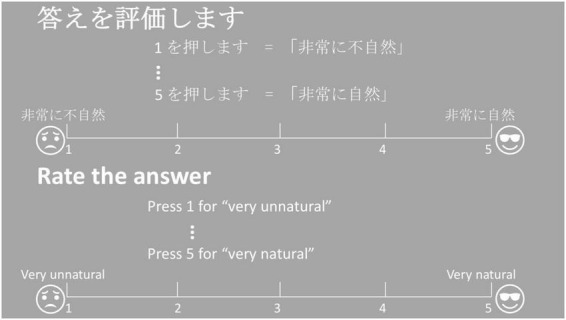
Judgment of JP and CA by sensory modality and focus type.

Figure 4 summarizes the responses made by the two participant groups. Participants’ responses were significantly influenced by *Focus*, the interaction between *Focus* and *Pitch Accent*, the interaction between *Participant Group* and *Focus* as well as the three-way interaction between *Participant Group*, *Focus* and *Sensory Modality* (Table 4).

**TABLE 4:** CLMM results on participants’ responses.

	Estimate	SE	*Z*	*p*
**Auditory-only and visual-auditory stimuli**				
Focus [BQCA]	−0.94114	0.12678	−7.424	1.14 × 10−13
Focus [BQNA]	−1.35843	0.12683	−10.71	2.00E−16
Accent [unaccented]	0.25473	0.11921	2.137	0.03261
Information [visual and auditory]	−0.023	0.0894	−0.257	0.79699
Participant group [JP]	0.17901	0.10323	1.734	0.08291
Focus [BQCA]: accent [unaccented]	−0.34391	0.1095	−3.141	0.00168
Focus [BQNA]: accent [unaccented]	−0.12322	0.10963	−1.124	0.26103
Focus [BQCA]: information [visual and auditory]	−0.19492	0.1659	−1.175	0.24004
Focus [BQNA] :information [visual and auditory]	0.05638	0.16527	0.341	0.73298
Information [visual and auditory]: participant group [JP]	0.06075	0.12637	0.481	0.6307
Focus [BQCA]: participant group [JP]	−0.63152	0.16048	−3.935	8.31E−05
Focus [BQNA]: participant group [JP]	−0.01127	0.1606	−0.07	0.94404
Focus [BQCA]: participant group [JP]	0.74665	0.23499	3.177	0.00149
Focus [BQNA]: information [visual and auditory]: participant group [JP]	−0.46017	0.23533	−1.955	0.05053
**Threshold coefficients:**				
1| 2	−2.5132	0.1127	−22.294	−0.001
2| 3	−0.6183	0.1081	−5.718	−0.001
3| 4	−0.3192	0.1079	−2.957	0.003
4| 5	1.6557	0.1108	14.944	−0.001
**Random effects**				
σ^2^	3.29			
t_00 Participant_	0.04			
t_00 StimuliNo._	0.07			
ICC	0.03			
N _StimuliNo._	28			
N _Participant_	60			
Observations	6720			

CLMM results of both auditory-only and visual-auditory stimuli. Mathematically, the threshold coefficients “1| 2” corresponds to P(*Y* ≥ 1), and can be interpreted as the log of odds of getting a response of 1 versus getting a score of 2 or above. Similarly, 2 | 3 corresponds to P(*Y* ≥ 2), and can be interpreted as the log of odds of getting a score of 1 or 2 versus getting a score of 3 or above. The rest threshold coefficients can be interpreted likewise. The negative coefficients of the fixed effects indicate certain fix effects or interaction correspond to lower response scores and the positive indicate that they correspond to higher response scores.

According to the *post hoc* comparison, significant differences between the two sensory modalities were only found in the mismatched trials in JP. To be specific, when answers carried contrastive focus, visual-auditory stimuli led to significantly better judgment of naturalness than the auditory-only stimuli (*p* < 0.001), indicating the facilitating effects of visual cues; by contrast, when answers carried non-contrastive narrow focus only, it was the auditory-only stimuli that led to significantly higher scores in the JP group (*p* < 0.05), indicating inhibiting effects of visual cues. When presented with visual-auditory stimuli, the JP group scored significantly higher than the CA group when answers carried broad and contrastive focus (*p*s < 0.05); when presented with auditory-only stimuli, however, the CA group scored significantly higher than the JP group when the answer carried contrastive focus (*p* < 0.005). Within each participant group, the scores of broad focus were significantly higher than the scores of the contrastive and non-contrastive narrow focus in both sensory modalities (Table 4). It is worth mentioning, however, such significance may be due to the difference in matchedness rather than the focus types, that is, mismatched trials only existed in conditions involving narrow focus. The significantly higher scores of broad focus was essentially a bias toward judging the stimuli as natural rather than unnatural. In addition, within the JP group, when the stimuli were presented in visual-auditory modality, the score of contrastive focus was also significantly higher than the score of non-contrastive narrow focus (*p* < 0.001).

### 3.2. Acoustic and visual cues

***BQBA***. When both visual and auditory cues were presented, the judgmentof naturalness in the JP group was only significantly influenced by the average intensity of the object syllable, but not other cues (Table 5). The positive estimated coefficients suggest that the louder the on-focus syllables were, the more likely the question-answer pairs would be judged as natural by the JP participants.

**TABLE 5:** CLMM results of visual-auditory stimuli with broad focus.

	Visual and auditory cues								Visual cues only							
	***JP***				***CA***				***JP***				***CA***			
	**Esti-** **mate**	**SE**	***Z***	***p***	**Esti-** **mate**	**SE**	***Z***	***p***	**Esti-** **mate**	**SE**	***Z***	***p***	**Esti-** **mate**	**SE**	***Z***	***p***
Average *f*_0_ of objects	−0.0002361	0.0036637	−0.064	0.948625												
Average Intensity of objects	0.1260095	0.0377436	3.339	**0.000842**	0.154208	0.037932	4.065	**4.80 × 10^–5^**								
Word accent [unaccented]					0.431525	0.142781	3.022	**0.002509**					0.3263	0.1606	2.032	**0.0422**
*F*_0_ range of objects					0.012509	0.003444	3.632	**0.000281**								
Max *f*_0_ of objects					−0.128596	0.078223	−1.644	0.100181								
Head displacement - left					−28.05811	15.285042	−1.836	0.066408								
Head displacement - right									−15.419	28.088	−0.549	0.583				
head displacement - up					18.539413	9.84961	1.882	0.059802								
Head displacement - down									−15.468	9.808	−1.577	0.115	1149.5632	193.0335	5.955	**2.60 × 10** ^–^ **^9^**
Left eyebrow displacement									1671.443	111.923	14.934	**<2 × 10** ^–^ **^16^**	−47.7696	31.3933	−1.522	0.1281
Head velocity - down													−37.1044	6.4639	−5.74	**9.45 × 10** ^–^ **^9^**
Left eyebrow velocity									−57.442	3.833	−14.986	**<2 × 10** ^–^ **^16^**				
Right eyebrow displacement													30.7615	27.9491	1.101	0.2711
**Threshold coefficients:**																
1| 2	4.562	1.937	2.355	**0.019**	8.01	2.275	3.521	**<0.001**	−2.9953	0.2119	−14.138	**<0.001**	−2.8039	0.2341	−11.979	**<0.001**
2| 3	6.576	1.947	3.377	**0.001**	9.868	2.286	4.317	**<0.001**	−1.1878	0.1736	−6.841	**<0.001**	−1.1074	0.2069	−5.354	**<0.001**
3| 4	6.866	1.949	3.523	**<0.001**	10.178	2.288	4.448	**<0.001**	−0.9371	0.1722	−5.443	**<0.001**	−0.8281	0.205	−4.04	**<0.001**
4| 5	9.275	1.965	4.719	**<0.001**	12.57	2.306	5.451	**<0.001**	1.0762	0.1773	6.069	**<0.001**	1.2705	0.2077	6.116	**<0.001**
***Random Effects***																
σ2	3.29															
t00	1.22 _Participant_				0.51 _Participant_											
	0.12 _SentenceNo_				0.18 _SentenceNo_				0.01 _SentenceNo_				0.04 _SentenceN_o			
ICC	0.29				0.17				0				0.01			
N	14 _SentenceNo_															
	30 _Participant_															
***Observations***	840				840			840	840				840			

The results could interpreted like Table 4. Bold values represent significance.

In the CA group, significant effects were also found among the auditory cues of the object syllables only (Table 5). The larger the *f*_0_ range and the average intensity were, the more likely the question-answer pairs would be judged as natural by the CA participants. Also, the answers with unaccented words were significantly more likely to be judged as natural by the CA participants, but pitch accent pattern did not influence judgment in the JP group in the broad focus condition.

When visual cues were examined in separation, in the JP group, the displacement and the velocity of the left eyebrow during the on-focus periods contributed significantly to the participants’ judgmentof naturalness (Table 5). The coefficients suggest that the larger but slower left eyebrow movement would lead the question-answer pairs to be judged as more natural. In the CA group, the degree and velocity of downward head movement reached significance (Table 5). The larger and slower head nodding would lead the question-answer pairs to be judged as more natural.

***BQCA***. When both visual and auditory cues were presented, in the JP group, the judgmentof naturalness was still influenced by auditory cues only (Table 6). The negative coefficients suggested that the lower the average *f*_0_ and the smaller the minimum *f*_0_ difference between the neighboring time points were in on-focus object syllables, the significantly higher the JP participants scored on naturalness judgement, i.e., judging the question-answer pair as unnatural. In addition, the lower the intensity of the post-focus verb syllables were, the more likely the question-answer pairs were correctly judged as unnatural.

**TABLE 6:** CLMM results of visual-auditory stimuli with contrastive narrow focus.

	Visual and auditory cues								Visual cues only							
	***JP***				***CA***				***JP***				***CA***			
	**Esti-** **mate**	**SE**	**Z**	***p***	**Esti-** **mate**	**SE**	**Z**	***p***	**Esti-** **mate**	**SE**	**Z**	***p***	**Esti-** **mate**	**SE**	**Z**	***p***
Average *f*_0_ of objects	−0.0176	0.00524	−3.359	**0.000782**	−0.013369	0.004078	−3.279	**0.00104**								
Average intensity of objects	−0.07806	0.02667	−2.927	**0.003424**												
Min f0 of objects	−0.44776	0.17258	−2.594	**0.009473**												
Duration of objects					−0.003566	0.001528	−2.334	**0.01962**								
Mean intensity of objects					−0.115645	0.043796	−2.641	**0.00828**								
Head displacement - up													−0.5139	0.3154	−1.629	0.1033
Head displacement - down					26.594756	10.734783	2.477	**0.01323**					30.7421	13.0936	2.348	**0.0189**
Head displacement - right									33.33	19.745	1.688	0.0914				
Left eyebrow displacement									23.864	9.858	2.421	0.0155				
Right eyebrow displacement													48.8922	12.1121	4.037	5.42 **× 10**^–^**^5^**
***Threshold coefficients:***																
1| 2	−9.912	1.804	−5.495	**<0.001**	−13.579	2.432	−5.583	**<0.001**	−1.8588	0.3315	−5.608	**<0.001**	−1.4479	0.3562	−4.065	**<0.001**
2| 3	−7.48	1.772	−4.222	**<0.001**	−11.036	2.407	−4.586	**<0.001**	0.4966	0.3219	1.543	0.123	1.0719	0.3596	2.981	**0.003**
3| 4	−7.089	1.767	−4.011	**<0.001**	−10.725	2.404	−4.462	**<0.001**	0.8767	0.325	2.698	**0.007**	1.378	0.3635	3.79	**<0.001**
4| 5	−5.204	1.758	−2.96	**0.003**	−9.153	2.396	−3.82	**0.001**	2.738	0.3587	7.633	**<0.001**	2.94	0.396	7.425	**<0.001**
***Random Effects***																
σ2	3.29				3.29				3.29				3.29			
t00	0.99 _Participant_				0.64 _Participant_				0.92 _Participant_				0.65 _Participant_			
	0.00 _SentenceNo_				0.03 _SentenceNo_				0.01 _SentenceNo_				0.32 _SentenceNo_			
ICC					0.17				0.22				0.23			
N	14 _SentenceNo_				14 _SentenceNo_				14 _SentenceNo_				14 _SentenceNo_			
	15 _Participant_				15 _Participant_				15 _Participant_				15 _Participant_			
***Observations***	435				435				435				435			

The results could interpreted like Table 4. Bold values represent significance.

In the CA group, both the auditory and visual cues significantly influenced the judgmentof naturalness (Table 6). To be specific, an on-focus object syllable with shorter duration and lower *f*_0_ and intensity would lead to the question-answer pair to be judged as unnatural, and so would a larger downwards head moment.

When visual cues were examined in separation, the judgment by the JP group was also significantly influenced by the displacement of the left eyebrow (Table 6). The larger the displacement was, the more likely the stimuli were correctly judged as unnatural. The judgment by the CA group, by contrast, was influenced by the displacement of the right eyebrow and downwards head movement (Table 6). The larger the displacement or the movement was, the more likely the stimuli were correctly judged as unnatural.

***BQNA***. When both visual and auditory cues were presented, the judgmentof naturalness in the JP group was significantly influenced by the duration of the on-focus object syllables and the displacement of the left eyebrow (Table 7). A shorter object syllable and/or larger left eyebrow movement would lead to a higher score in JP judgement, that is, to have the stimuli correctly judged as unnatural.

**TABLE 7:** CLMM results of visual-auditory stimuli with non-contrastive narrow focus.

	Visual and auditory cues								Visual cues only							
	***JP***				***CA***				***JP***				***CA***			
	**Esti-** **mate**	**SE**	**Z**	***p***	**Esti-** **mate**	**SE**	**Z**	***p***	**Esti-** **mate**	**SE**	**Z**	***p***	**Esti-** **mate**	**SE**	**Z**	***p***
Duration of objects	−0.004382	0.001619	−2.706	**0.00681**	−0.003231	0.001762	−1.834	0.0666								
Average *f*_0_ of objects	−0.004062	0.004403	−0.923	0.35619	−0.002429	0.004256	−0.571	0.5682								
Max *f*_0_ of objects	0.149797	0.103727	1.444	0.1487												
Left eyebrow displacement	25.030831	12.418063	2.016	**0.04383**	−38.65353	28.97295	−1.334	0.1822	34.43	13.24	2.602	**0.00928**				
F0 range of verbs					−0.001595	0.001338	−1.192	0.2331								
Average intensity of verbs					−0.056157	0.02605	−2.156	**0.0311**								
Head displacement - left					1.202867	0.559346	2.15	**0.0315**								
Right eyebrow velocity					1.781649	0.993473	1.793	0.0729								
Right eyebrow displacement													5.94E+01	1.59E+01	3.729	**0.000192**
Head displacement - right													−2.37E+03	1.23E+02	−19.311	**<2 × 10** ^–^ **^16^**
Head velocity - right													8.10E+01	4.21E+00	19.257	**<2 × 10** ^–^ **^16^**
Head velocity - up													7.02E−03	9.55E−03	0.735	0.462
***Threshold coefficients:***																
1| 2	−3.1415	1.2962	−2.424	**0.015**	−6.237	1.874	−3.329	**0.001**	−0.9188	0.4133	−2.223	**0.026**	−1.1667	0.214	−5.451	**<0.001**
2| 3	−0.8931	1.286	−0.694	0.487	−3.994	1.857	−2.151	**0.031**	1.3239	0.4154	3.187	**0.001**	0.9413	0.2099	4.484	**<0.001**
3| 4	−0.5565	1.2855	−0.433	0.665	−3.694	1.855	−1.991	**0.047**	1.6577	0.4183	3.963	**<0.001**	1.2244	0.2139	5.724	**<0.001**
4| 5	1.8927	1.2999	1.456	0.145	−1.412	1.858	−0.76	0.447	4.096	0.4815	8.506	**<0.001**	3.4592	0.3089	11.198	**<0.001**
***Random effects***																
σ2	3.29				3.29				3.29				3.29			
t00	1.96 _Participant_				0.23 _Participant_				1.95 _Participant_				0.02 _SentenceNo_			
	0.00 _SentenceNo_				0.00 _SentenceNo_				0.08 _SentenceNo_							
ICC									0.38				0.01			
N	14 _SentenceNo_				14 _SentenceNo_				14 _SentenceNo_				14 _SentenceNo_			
	15 _Participant_				15 _Participant_				15 _Participant_				15 _Participant_			
***Observations***	405				405				405				405			

The results could interpreted like Table 4. Bold values represent significance.

In the CA group, the effects of the mean intensity of the post-focus verb syllables and the leftwards head movement were significant (Table 7). Lower post-focus intensity and larger leftwards head movement would lead to a significantly higher score, i.e., to have the stimuli correctly judged as unnatural.

When visual cues were examined in separation, it was still the displacement of the left eyebrow that reached significance in the JP group while in the CA group, the displacement of the right eyebrow and the degree and velocity of the rightwards head movement were significant (Table 7). To be specific, the larger displacement of right eyebrow and the smaller and quicker rightwards head movement were, the more likely the CA participants would judge the question-answer pairs as unnatural correctly.

## 4. Discussion

Japanese speakers did refer to visual cues when performing the judgmentof naturalness. However, the effects of visual cues were not significant in the broad focus trials with matched questions and answers, but were significant in the mismatched narrow focus trials, namely, the questions carried broad focus but the answers carried contrastive and non-contrastive narrow focus. However, such results do not contradict with Sekiyama’s studies on segmental perception (Sekiyama and Tohkura, 1991; Sekiyama, 1994; Sekiyama et al., 1995), but add to the evidence that Japanese speakers tend to use visual information when the auditory information is not clear enough. In the present study, to be specific, is when there was a mismatch in prosodies of questions and answers. Moreover, the integration of visual cues facilitated the processing of contrastive narrow focus but impeded the processing of non-contrastive narrow focus. To be specific, when presented with visual cues, JP tended to correctly judge the trials with questions eliciting broad focus and answers carrying contrastive focus as unnatural but incorrectly judge the trials with questions eliciting broad focus and answers carrying non-contrastive focus as natural.

These findings, together with the previous studies on McGurk Effects in Japanese suggest that generally there is a low rate of visual-auditory integration in Japanese-speaking population. It is possible that the widely reported connection between pitch and facial movements is over-ridden by the habit of relying on auditory cues only developed in learning Japanese (Sekiyama and Burnham, 2008). We propose that the implicitness of Japanese culture in which people are less encouraged to make large facial movements (Matsumoto and Ekman, 1989; Matsumoto, 1992) may also reduce Japanese speakers’ attention to visual cues and hence their visual-auditory integration abilities in speech perception. This helps to explain why the relatively weak and complex prosodic system in Japanese still did not lead to stronger use of visual cues in prominence perception. The face masks in the present study may have also added to the difficulty in the use of visual cues but to a limited degree since prosodic focus has been found to be signaled mainly by eyebrows and head, which were not affected to a great extent after wearing a mask (e.g., Krahmer and Swerts, 2007).

Very limited foreign language effects were found in the integration of visual cues by the Cantonese participants. No significant differences were found between the auditory-only and visual-auditory modalities among Cantonese participants, regardless of the focus types or the matchedness of the question-answer pairs. When both visual and auditory cues were analyzed in detail, the visual cues in the CA group showed significant effects only when there was a mismatch between the question-answer pair (i.e., carrying contrastive and non-contrastive narrow focus), much like the JP counterparts except that the lack of visual cues significantly affected the judgment for answers carrying contrastive focus in the JP group. Therefore, with regard to H1, our results confirm that both native and non-native listeners used visual cues in the perception of focal prominence in Japanese when there was a mismatch in question-answer pairs, but the Cantonese-native learners of Japanese did not rely more on the visual cues than the native speakers. Although previous studies show that Cantonese speakers do integrate visual cues in lexical tone perceptions in continuous speech (Burnham et al., 2001a,^2022^), it may not necessarily be the case in focus perception of a foreign language.

With regard to the second research question, the influences of auditory cues were significant in both groups across three focus types, but the influences of visual cues were only significant on the judgmentof non-contrastive focus in both groups and the contrastive focus in the CA group. Therefore, it may be concluded that auditory cues were more influential in Japanese focus perception. The specific auditory cues that contributed significantly to perception also varied across participant groups and focus types. Both the JP and CA participants used the acoustic cues of the on-focus and post-focus syllables, and the Cantonese participants did not rely systematically on f0 nor duration cues unlike when perceiving focus in Cantonese. Therefore, H2 is supported, and overall visual-auditory focus perception in Japanese is a complex process for both native and non-native speakers.

Finally, H3 is also partially confirmed as Japanese speakers did show a preference to left eyebrow movements, echoing Krahmer and Swerts (2007), whereas Cantonese speakers tended to rely more on head movement in focus perception. Cantonese participants also used more visual cues in a less systematic way than Japanese speakers especially when the questions and answers were mismatched. In other words, the visual cues and the direction of their movements used by CA differed between focus conditions. The complex patterns observed in the CA group might be due to the participants’ unfamiliarity with Japanese facial expressions. After all, facial expressions are highly culture-specific (Ekman et al., 1987; Matsumoto and Ekman, 1989; Matsumoto, 1992; Biehl et al., 1997). It is well established in these studies that participants from different cultures or even speech communities pick up on different cues in emotion and attitudes. Therefore, JP might be more familiar with facial expressions made by speakers of their own culture and hence could pick up some visual cues in a relatively stable manner (i.e., the movements of left eyebrows according to the present finding). CA, by contrast, might attend to both the eyebrow and head movements, and hence showed a non-systematic use of the visual cues. Another point worth noting is that larger rather than smaller and slower rather than quicker movements significantly facilitated naturalness judgement, showing that only exaggerated facial and head movements would be used by the listeners in prosodic perception by both the native and non-native speakers. Such findings are in line with segmental perception in Japanese, namely, visual and auditory information is integrated when the speakers make exaggerated articulatory movements (Sekiyama, 1994; Sekiyama et al., 1995). It may be interpreted as the evidence supporting the limited facial movement hypothesis, that is, the weak McGurk Effects observed in Japanese speech may be due to the relatively limited facial and head movements Japanese speakers use when speaking, and the facial masks in the present study may have further constrained their movements. Kinematic analyses of facial and head movements of speakers cross cultures and nations are required to test this hypothesis in future studies.

In conclusion, the present study tested multi-sensory perception of focal prominence in Japanese by native and non-native listeners. An integration of audio and visual cues was only observed when there was a mismatch in the auditory information. Furthermore, the non-native Japanese learners with Cantonese as native languages did not show obviously enhanced integration either. Such lack of integration may be due to their unfamiliarity with Japanese facial expressions and the limited use of facial and head movements in Japanese speech communication overall. With the lower face masked, the upper face and head together can signal prosodic focus in Japanese, but the preference to the left facial areas was only observed in the native Japanese speakers. The present findings indicate that the integration of visual cues in prosodic perception is more specific to language and culture, which is different from the commonly observed relationship between f0 and head/face movements in speech production. This indication calls for more cross-linguistic tests.

## Data availability statement

The raw data supporting the conclusions of this article will be made available by the authors, without undue reservation.

## Ethics statement

The studies involving humans were approved by the PolyU Institutional Review Board. The studies were conducted in accordance with the local legislation and institutional requirements. The participants provided their written informed consent to participate in this study.

## Author contributions

YZ: conceptualization, original draft preparation, data analysis, and review and editing. XC: original draft preparation, experimental design, data curation, data analysis, and review and editing. SC: conceptualization, experimental design, funding acquisition, and review and editing. YM: experimental design and data curation. AL: conceptualization, experimental design, and review and editing. All authors contributed to the article and approved the submitted version.
