# Supplementary material for: Visual-auditory perception of prosodic focus in Japanese by native and non-native speakers
Source: Front Hum Neurosci. 2023 Sep 21;17:1237395. doi: 10.3389/fnhum.2023.1237395 (PMC10552536; doi:10.3389/fnhum.2023.1237395)
Supplement: Supplementary file 1 [file Data_Sheet_1.PDF]

Please try to remember these pictures and corresponding sentences.

1

久保田さんは本を取った。

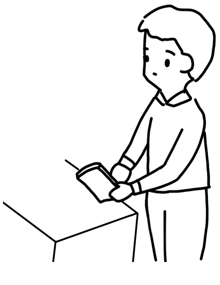

久保田さんは何を取ったの？

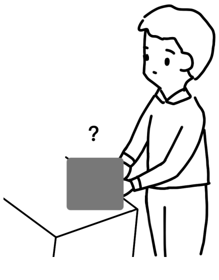

久保田さんは傘を取ったの？

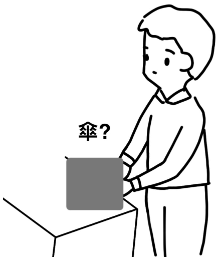

2

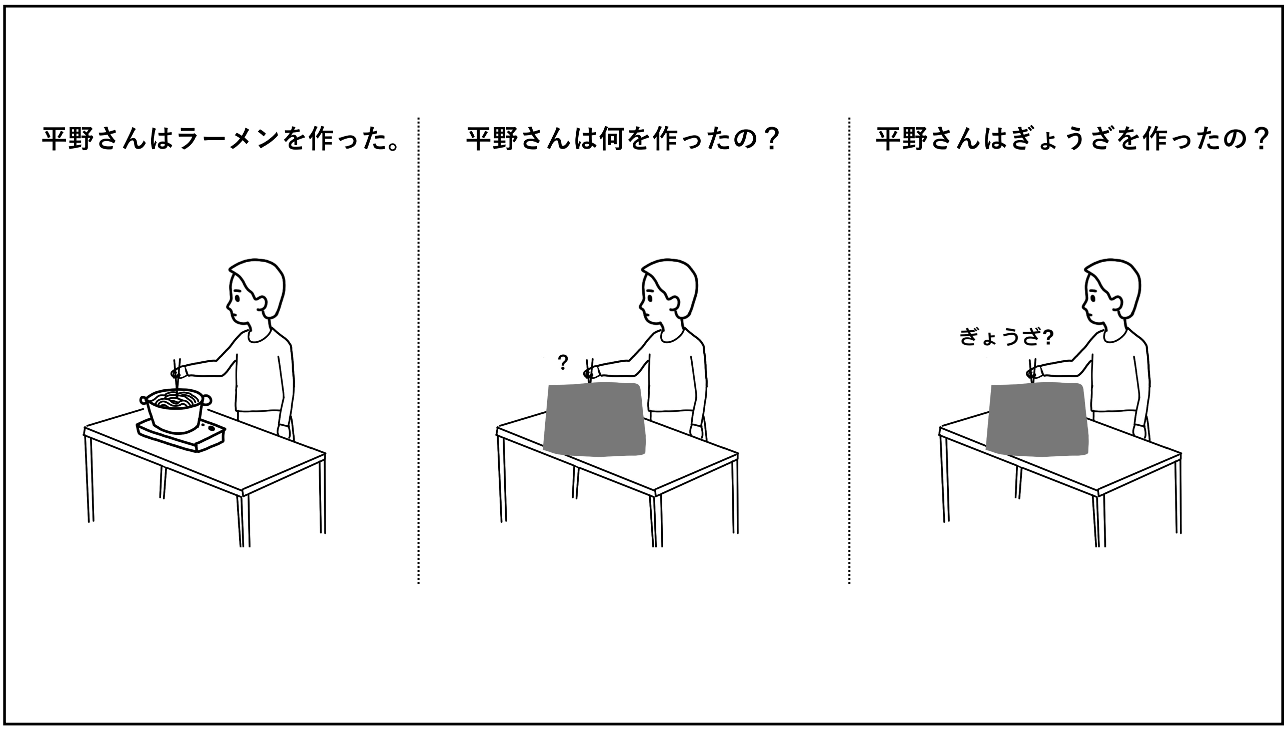
平野さんはラーメンを作った。
平野さんは何を作ったの？
?
平野さんはぎょうざを作ったの？
ぎょうざ?

3

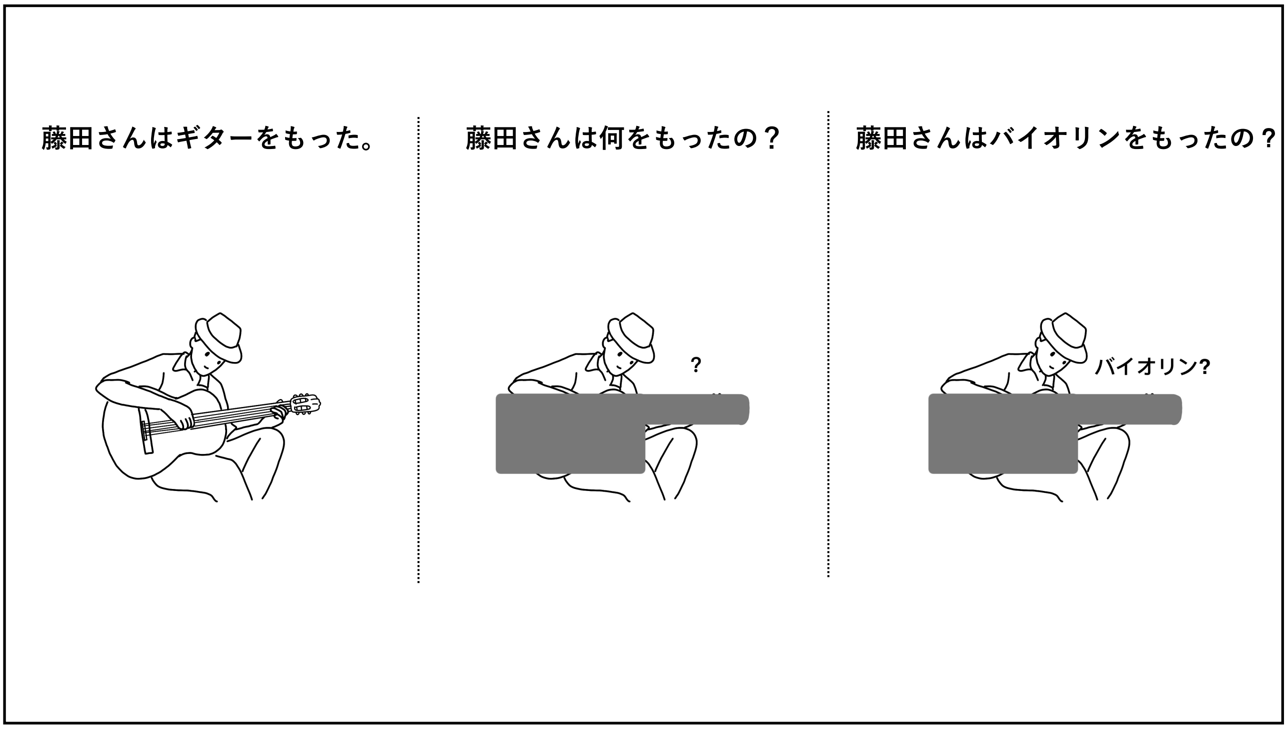
藤田さんはギターをもった。
藤田さんは何をもったの？
?
藤田さんはバイオリンをもったの？
バイオリン?

4

酒井さんは猫を飼った。

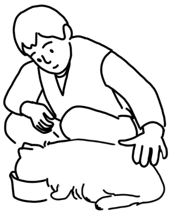

酒井さんは何を飼ったの？

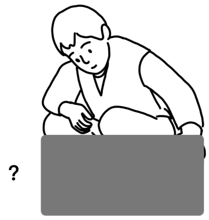

酒井さんは犬を飼ったの？

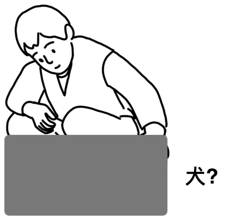

5

田村さんはワインを飲んだ。

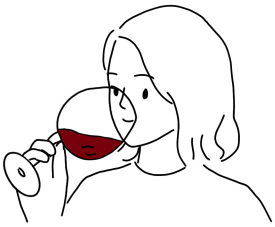

田村さんは何を飲んだの？

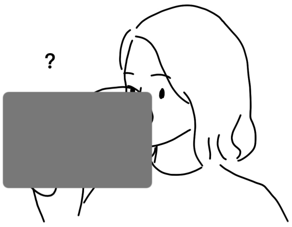

田村さんはジュースを飲んだの？

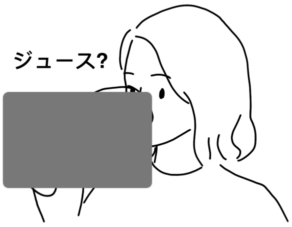

6

柴田さんは授業を受けた。

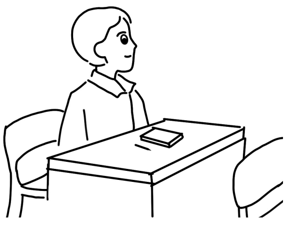

柴田さんは何を受けたの？

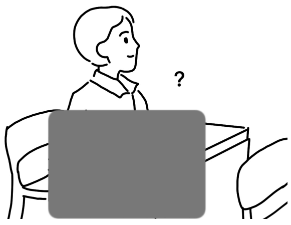

柴田さんはインタビューを受けたの？

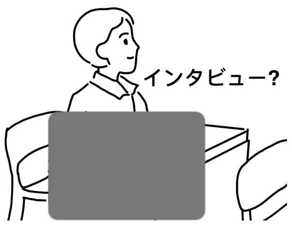

7

森田さんはキノコを採った。

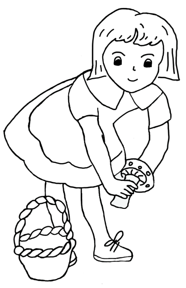

森田さんは何を採ったの？

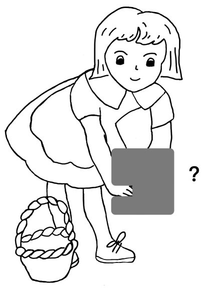

森田さんはいちごを採ったの？

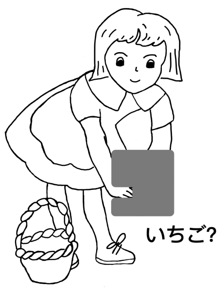

8

小山さんはサラダを残した。

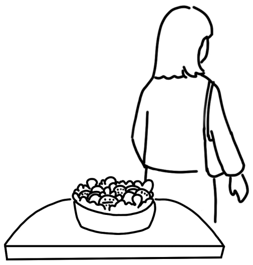

小山さんは何を残したの？

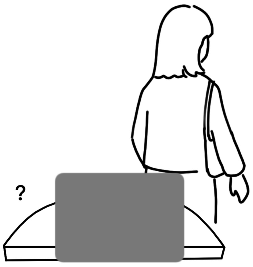

小山さんは肉を残したの？

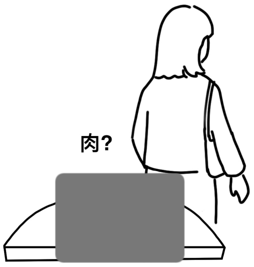

9

加藤さんはトマトを投げた。

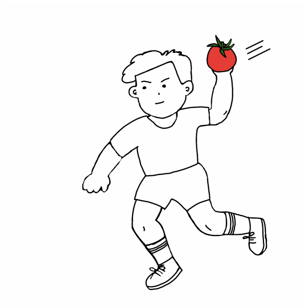

加藤さんは何を投げたの？

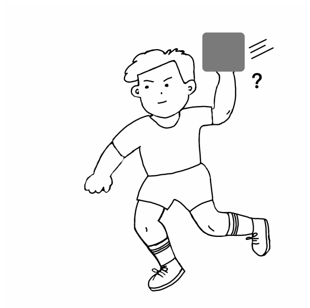

加藤さんはたまごを投げたの？

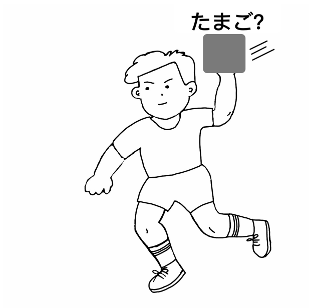

10

田口さんはデータを集めた。

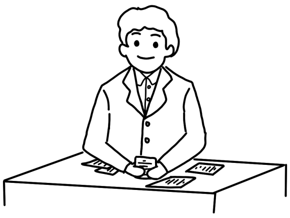

田口さんは何を集めたの？

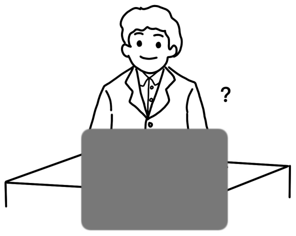

田口さんは絵を集めたの？

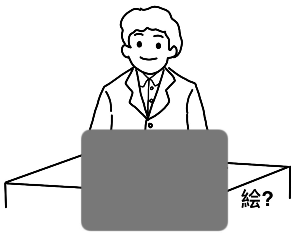

11

秋田さんは生徒を待った。

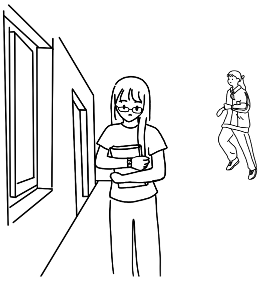

秋田さんは誰を待ったの？

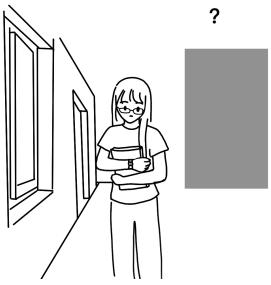

秋田さんは先生を待ったの？

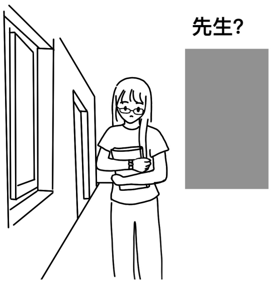

12

香川さんはたまごを炒めた。

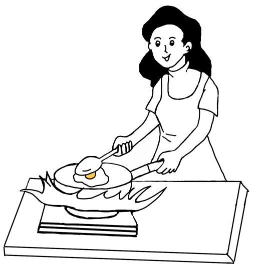

香川さんは何を炒めたの？

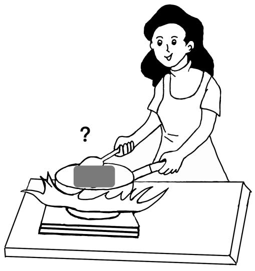

香川さんは野菜を炒めたの？

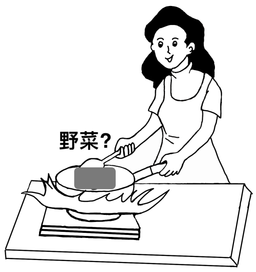

13

高野さんはビールを冷やした。

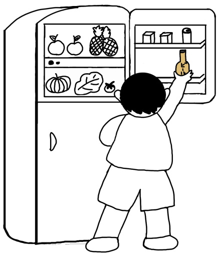

高野さんは何を冷やしたの？

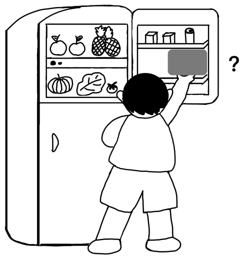

高野さんはジュースを冷やしたの？

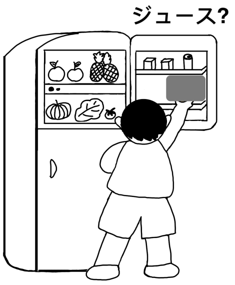

14

永井さんは井戸を掘った。

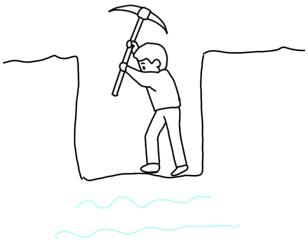

永井さんは何を掘ったの？

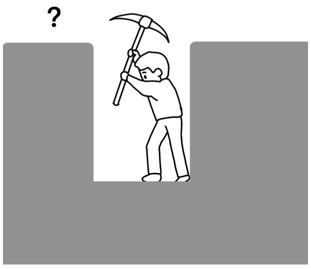

永井さんはあなを掘ったの？

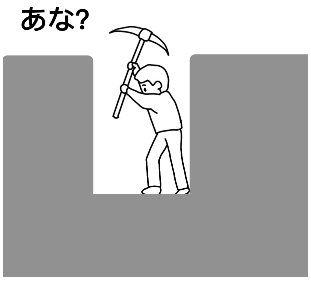

15

藤井さんはかばんを探した。

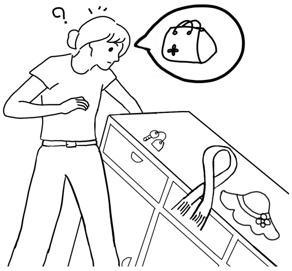

藤井さんは何を探したの？

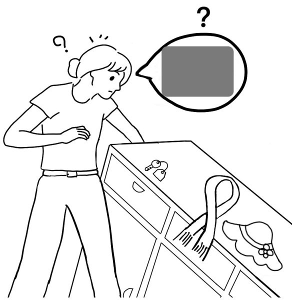

藤井さんは眼鏡を探したの？

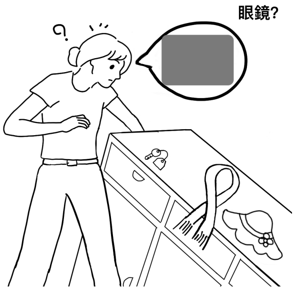

16

山田さんは魚を釣った。

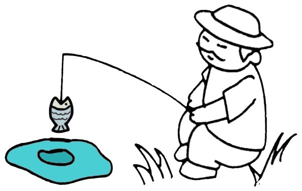

山田さんは何を釣ったの？

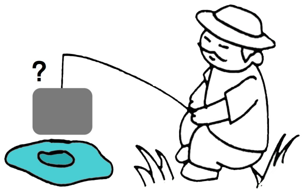

山田さんは海老を釣ったの？

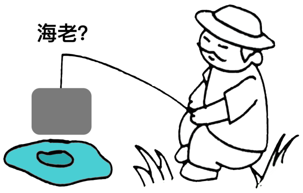

17

村田さんは畳を拭いた。

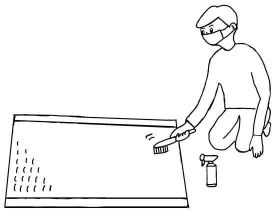

村田さんは畳を拭いたの？

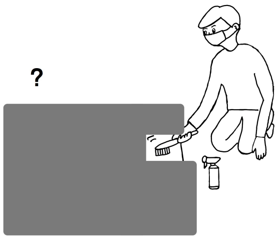

村田さんは窓を拭いたの？

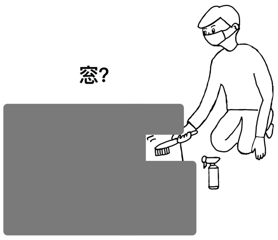

18

吉田さんはボタンを押した。

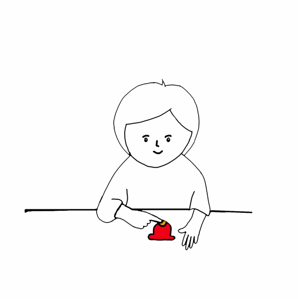

吉田さんは何を押したの？

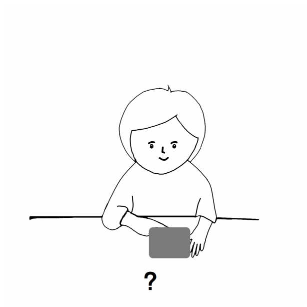

吉田さんはキーボードを押したの？

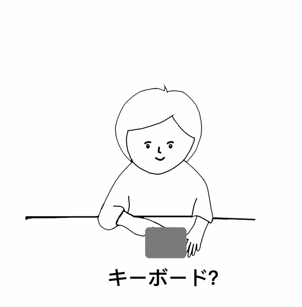

19

小松さんは指輪を忘れた。

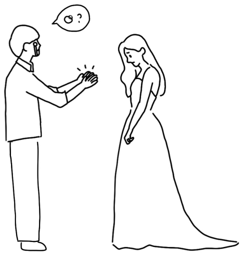

小松さんは何を忘れたの？

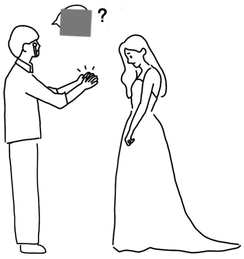

小松さんは携帯を忘れたの？

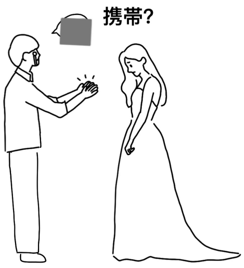

20

田中さんは時計を置いた。

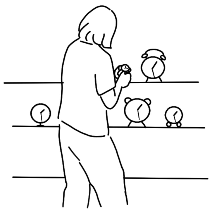

田中さんは何を置いたの？

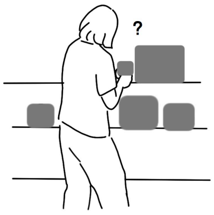

田中さんはおもちゃを置いたの？

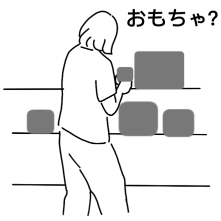

21

鈴木さんは医者を呼んだ。

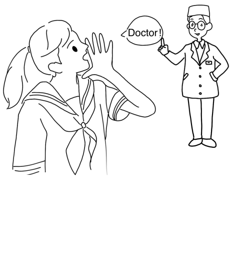

鈴木さんは誰を呼んだの？

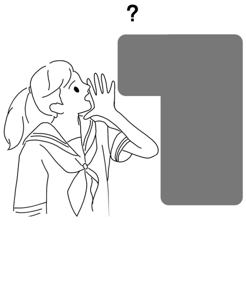

鈴木さんは先生を呼んだの？

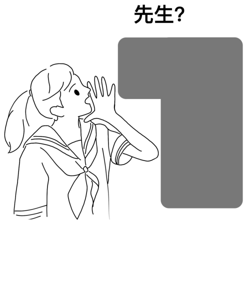

22

池田さんは車を洗った。

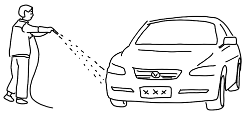

池田さんは何を洗ったの？

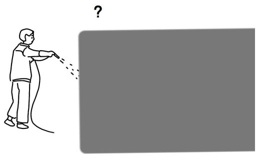

池田さんはタオルを洗ったの？

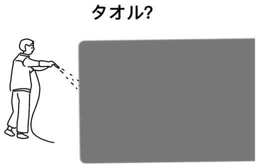

23

佐々木さんは写真を消した。

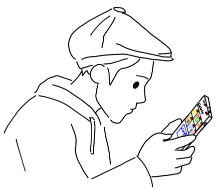

佐々木さんは何を消したの？

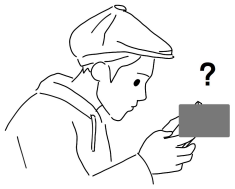

佐々木さんはデータを消したの？

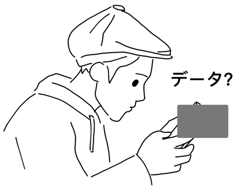

24

伊藤さんは勉強を始めた。

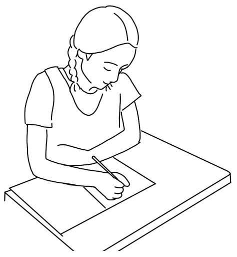

伊藤さんは何を始めたの？

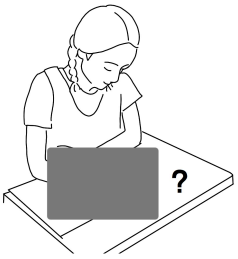

伊藤さんはゲームを始めたの？

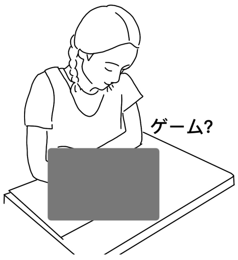

25

林さんはピアノを売った。

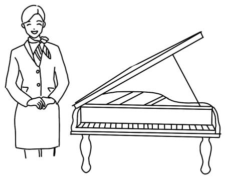

林さんは何を売ったの？

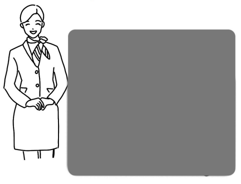

林さんはテーブルを売ったの？

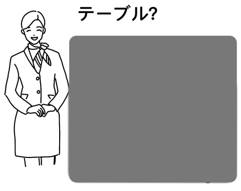

26

菊池さんは鏡を買った。

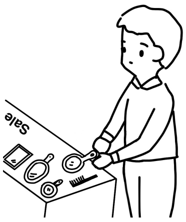

菊池さんは何を買ったの？

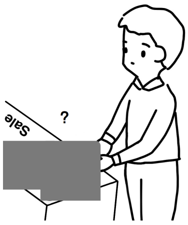

菊池さんはカメラを買ったの？

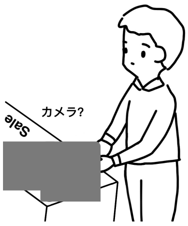

27

小島さんは息子を追った。

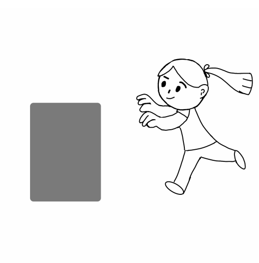

小島さんは誰を追ったの？

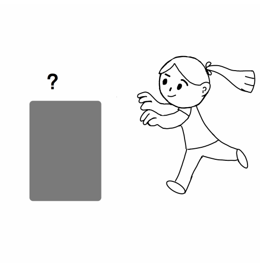

小島さんは娘を追ったの？

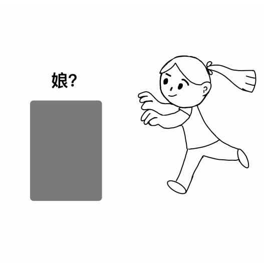

28

前田さんは鍵を無くした。

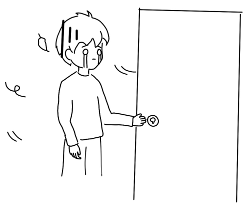

前田さんは何を無くしたの？

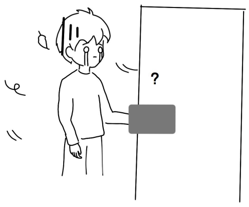

前田さんはカードを無くしたの？

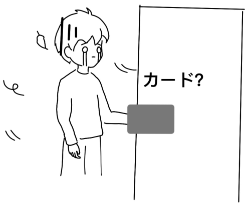

29

The following pictures and sentences do not need to be memorized, but please familiarize yourself with them if you have time.

30

小野さんは荷物を運んだ。

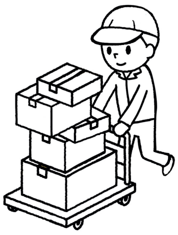

小野さんは何を運んだの？

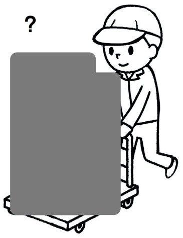

小野さんは石を運んだの？

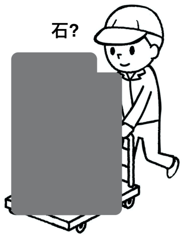

小野さんは荷物をどうしたの？

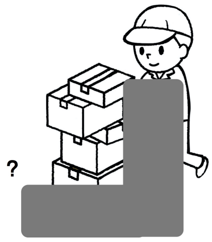

小野さんは荷物を整理したの？

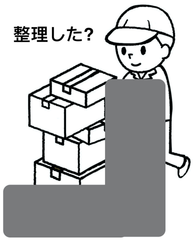

31

武田さんはタバコを吸った。

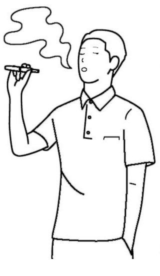

武田さんは何を吸ったの？

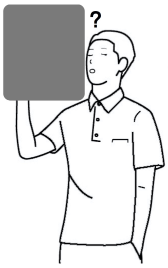

武田さんは空気を吸ったの？

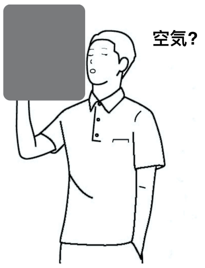

武田さんはタバコをどうしたの？

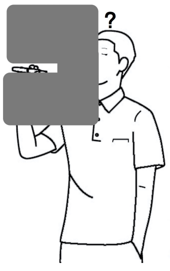

武田さんはタバコを捨てたの？

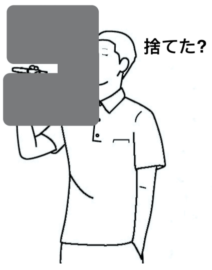

32

新井さんはパンを食べた。

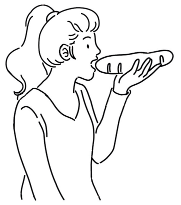

新井さんは何を食べたの？

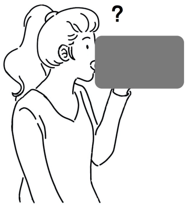

新井さんはケーキを食べたの？

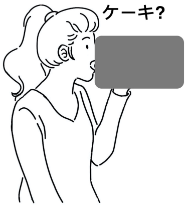

新井さんはパンをどうしたの？

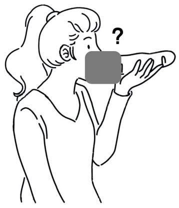

新井さんはパンを買ったの？

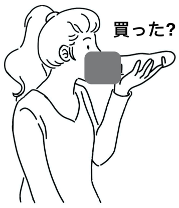

33

野口さんは歯を磨いだ。

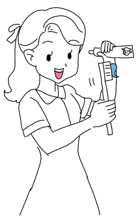

野口さんは何を磨いだの？

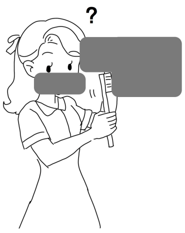

野口さんは靴を磨いだの？

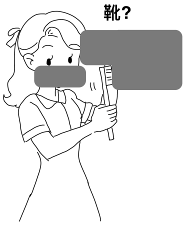

野口さんは歯をどうしたの？

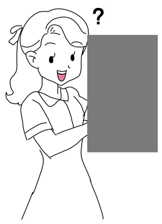

野口さんは歯を抜いたの？

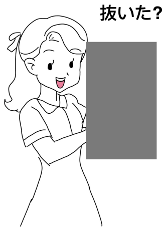

34

高木さんは牛乳を入れた。

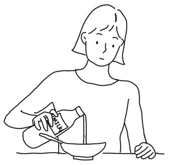

高木さんは何を入れたの？

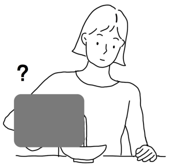

高木さんはお湯を入れたの？

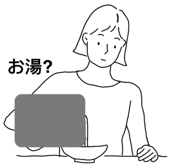

高木さんは牛乳をどうしたの？

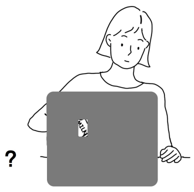

高木さんは牛乳を温めた？

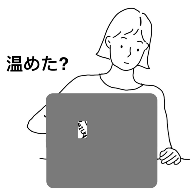

35

木下さんはガラスを割った。

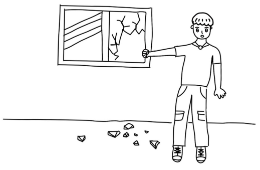

木下さんは何を割ったの？

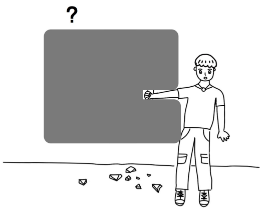

木下さんは皿を割ったの？

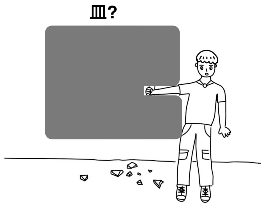

木下さんはガラスに何をしたの？

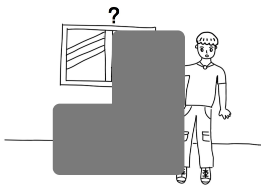

木下さんはガラスを叩いたの？

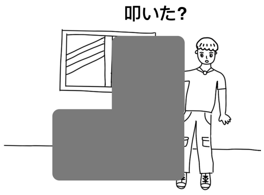

36

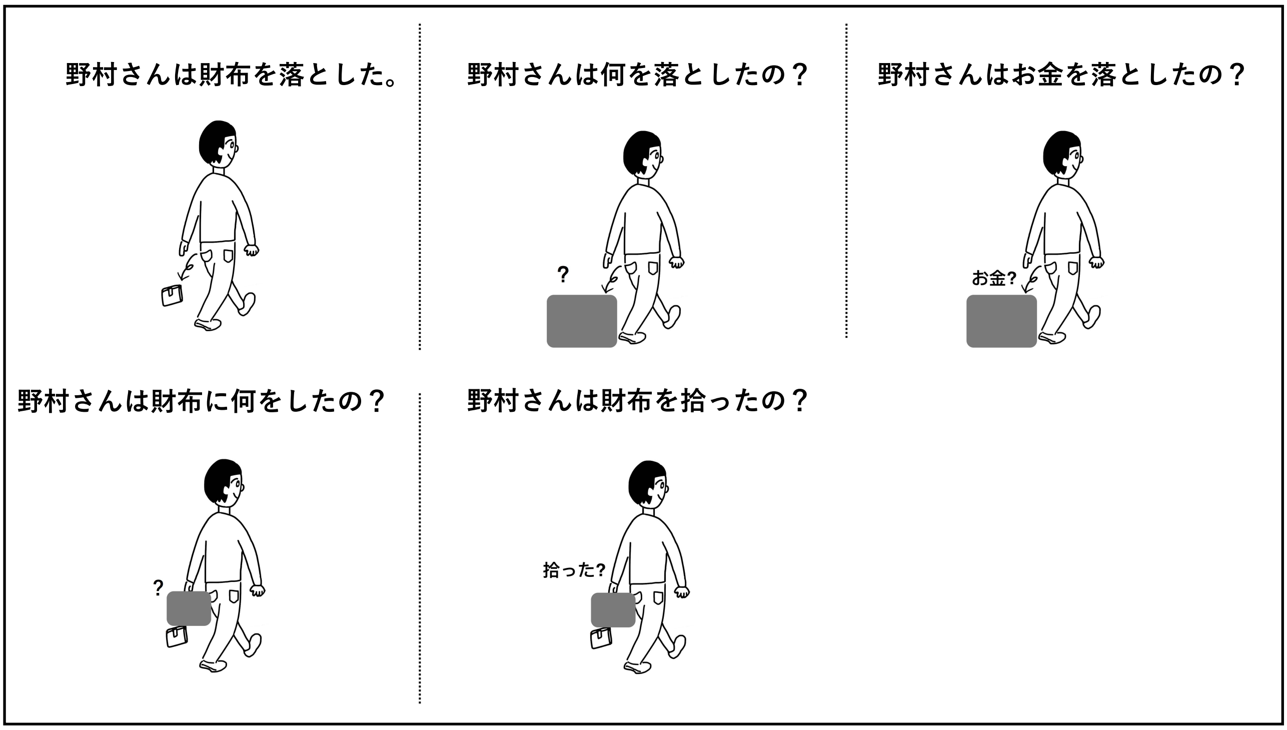
野村さんは財布を落とした。
野村さんは何を落としたの？
?
野村さんはお金を落としたの？
お金?
野村さんは財布に何をしたの？
?
野村さんは財布を拾ったの？
拾った?

37

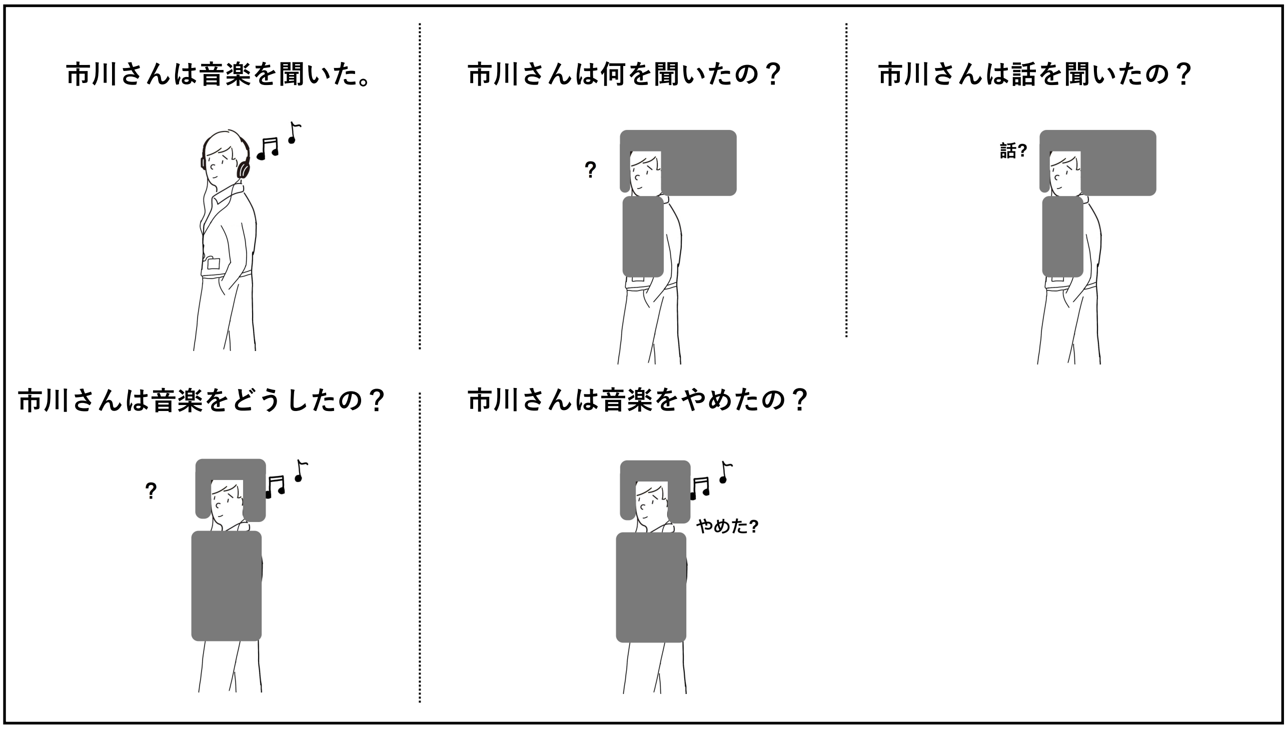
市川さんは音楽を聞いた。
市川さんは何を聞いたの？
?
市川さんは話を聞いたの？
話?
市川さんは音楽をどうしたの？
?
市川さんは音楽をやめたの？
やめた?

38

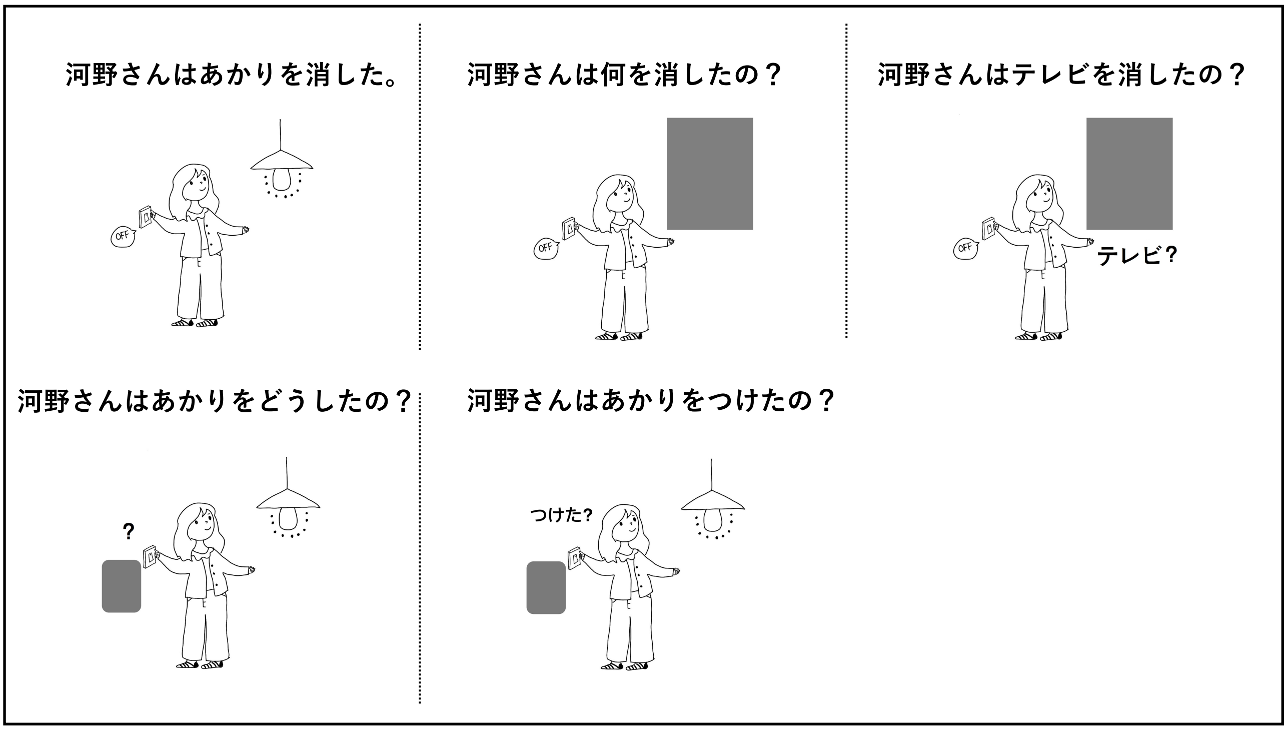
河野さんはあかりを消した。
OFF
河野さんは何を消したの？
OFF
河野さんはテレビを消したの？
OFF
テレビ?
河野さんはあかりをどうしたの？
?
河野さんはあかりをつけたの？
つけた?

39
